# Predictor Factors of Perceived Health in Family Caregivers of People Diagnosed with Mild or Moderate Alzheimer’s Disease

**DOI:** 10.3390/ijerph16193762

**Published:** 2019-10-07

**Authors:** María Dolores Ruiz-Fernández, José Manuel Hernández-Padilla, Rocío Ortiz-Amo, Cayetano Fernández-Sola, Isabel María Fernández-Medina, José Granero-Molina

**Affiliations:** 1 Department of Nursing Science, Physiotherapy and Medicine, University of Almeria, 04120 Almeria, Spain; mrf757@ual.es (M.D.R.-F.); 29rocii@gmail.com (R.O.-A.); cfernan@ual.es (C.F.-S.); isabel_medina@ual.es (I.M.F.-M.); jgranero@ual.es (J.G.-M.); 2 Adult, Child and Midwifery Department, School of Health and Education, Middlesex University, London NW4 4BT, UK; 3 Facultad de Ciencias de la Salud, Universidad Autónoma de Chile, 4810101 Temuco, Chile

**Keywords:** Alzheimer’s disease, nursing, caregiver, risk, protection, perceived health, mental health problems

## Abstract

Caring for a person diagnosed with Alzheimer’s disease has a negative impact on family caregivers’ psychological health. This study examined the factors related to ‘perceived health’ and ‘presence of new-onset mental health problems’ in family caregivers of people diagnosed with mild and moderate Alzheimer’s disease. A cross-sectional observational study carried out in Almeria’s Healthcare District (Spain). A total of 255 family caregivers (42.4% cared for people with mild Alzheimer’s disease and 57.6% cared for people with moderate Alzheimer’s disease) participated in the study from January to December 2015. Mainly, caregivers were women (81.5% in the mild Alzheimer’s disease group and 88.4% in the moderate Alzheimer’s disease group), and their average age was 56.54 years (standard deviation (SD) = 13.13) and 54.47 years (SD = 11.71), respectively. Around 47% of the caregivers had been caring for the person with Alzheimer’s between two and five years. The Goldberg General Health Questionnaire was used to measure perceived health and the presence of new-onset mental health problems. An exploratory descriptive analysis and a multivariate logistic regression analysis were conducted. For caregivers of people with mild Alzheimer’s disease, ‘perceived health’ was related to ‘perceived social support’ (*r* = −0.21; *p* = 0.028), ‘person’s level of dependency’ (*r* = −0.24, *p* = 0.05), ‘severity of the person’s neuropsychiatric symptoms’ (*r* = 0.22; *p* = 0.05), and ‘caregiver’s emotional distress in response to the person’s neuropsychiatric symptoms’ (*r* = 0.22; *p* = 0.05). For caregivers of people with moderate Alzheimer’s disease, ‘perceived health’ was related to ‘perceived social support’ (*r* = −0.31; *p* ˂ 0.01), ‘presence of neuropsychiatric symptoms’ (*r* = 0.27, *p* = 0.01), ‘severity of the person’s neuropsychiatric symptoms’ (*r* = 0.32, *p* = 0.01) and ‘caregiver’s emotional distress in response to the person’s neuropsychiatric symptoms’ (*r* = 0.029; *p* = 0.01). The presence of new-onset mental health problems was detected in 46.3% (*n* = 50) of caregivers of people with mild Alzheimer’s and 61.9% (*n* = 91) of caregivers of people with moderate Alzheimer’s. When people are diagnosed with mild Alzheimer’s disease, intervention programs for caregivers should aim to regulate emotions and promote positive coping strategies. When people are diagnosed with moderate Alzheimer’s disease, intervention programs for caregivers must allow them to adapt to caregiving demands that arise with the progression of Alzheimer’s disease.

## 1. Introduction

In recent years, the elderly population has grown at an accelerated pace due to increased life expectancy [1]. This is often linked to more people suffering from chronic diseases such as dementias [2]. It is estimated that around fifty million people worldwide are affected by some type of dementia [3]. Currently, approximately eight hundred thousand people suffer from some type of dementia in Spain, of which around 60%–80% correspond to Alzheimer’s disease [4].

Alzheimer’s disease (AD) is a neurodegenerative disease with an insidious onset and progressive course that is characterized by the deterioration of cognitive abilities and the development of behavioral disorders [5]. Broadly speaking, AD has three major stages: Early stage (persons diagnosed with mild AD), middle stage (persons diagnosed with moderate AD), and late stage (persons diagnosed with advanced AD). In the early stage, a gradual deterioration of episodic memory is observed along with the deficit of other cognitive abilities [6]. In the middle stage, all cognitive aspects of the persons begin to progressively fail. Emotional and social changes are also accentuated and aggravated while persons also begin to become dependent for basic activities of daily life [7]. In the late stage, cerebral symptoms worsen, and all intellectual faculties of the persons are affected, which leaves the person with a severe and serious dependency that ends in death [8].

From the beginning of the disease, people with AD need constant attention and supervision when performing all basic activities of their daily lives [9]. In most cases, the attention, support and care that people with AD require comes from their relatives [10,11]. Currently, families are getting smaller [12], so the caregiver’s role is assumed by only one person who bears most of the responsibility and overload that comes with the physical and emotional care of a person diagnosed with AD [13]. The relative who assumes most of the responsibility of caring for the person with AD is defined as a family caregiver [14]. Generally, family caregivers tend to be middle-aged (40–65 years-old) women (wife or daughter) [15], with a medium education level and who live in the person’s home [16]. This family caregiver role is often ascribed to women as part of an internalized feeling of obligation [15].

Caring for people with AD causes chronic stress and psychological distress in family caregivers, affecting their quality of life from the beginning of the disease [17]. Family caregivers are forced to modify their lifestyle and habits to adapt to their new role [18,19]. Family caregivers are obliged to perform actions of physical, psychological and social care in order to meet the needs of the person being cared for [20]. Most caregivers are dissatisfied or very dissatisfied with their general health, even if they are caring for family members diagnosed with mild AD [21]. They perceive that their health is very poor from the beginning of their relative’s illness [22].

Pearlin (1991) described a series of contextual and modulating factors that predict the negative impact of care on family caregivers’ health [23]. Among the contextual factors, the bond, the degree of kinship with the person cared for and the hours of daily care stand out [24]. The family caregiver’s social support and coping mechanisms are modulating factors that help them to reduce the impact of care [25,26]. Other studies have determined that in people diagnosed with AD, family caregivers’ overload is mainly related to the neuropsychiatric symptoms of the person and their level of dependency to perform basic activities in their daily lives [27]. Other characteristics of the caregiver such as gender, age, education level and emotional well-being, as well as the progression of the disease and the time spent caring for the person, affect the caregivers’ burden [28]. The continuous coexistence between both within the same household increases the levels of subjective perception and therefore the levels of stress in the caregiver [29].

To prevent or alleviate caregivers’ overload, improve their well-being, and optimize their coping strategies while caring for a relative with AD, it is important to implement intervention therapies from the earliest stage of the disease [30]. Family care in the early or moderate stages of the disease could improve family caregivers’ perceived health and avoid new-onset mental health problems and overload [31]. However, the factors that specifically affect family caregivers’ perceived health in the different stages of the person’s disease are not known [32].

Caring for a person diagnosed with AD has a negative impact on family caregivers’ quality of life and psychological health, causing an overload that culminates in caregiver burnout [33,34]. However, the factors that specifically affect family caregivers’ perceived health in the different stages of the person’s disease are not known. Therefore, the objective of this study was to determine the factors related to ‘perceived health’ and ‘presence of new-onset mental health problems,’ focusing in family caregivers of people diagnosed with mild or moderate Alzheimer’s disease.

## 2. Material and Methods

### 2.1. Design

This was a cross-sectional observational study. The Strengthening the Reporting of Observational Studies in Epidemiology (STROBE) statement recommendations were followed.

### 2.2. Participants

The study participants were family caregivers of people diagnosed with early or moderate AD. The family caregivers lived in different areas of Almeria’s Healthcare District (Spain). In order to recruit the study’s participants, the case manager nurses’ caregiver database was used in order to identify family caregivers of people diagnosed with AD. This database is included in the DIRAYA software, which is an electronic platform that allows healthcare professionals to manage healthcare histories of all users in the Andalusian Health System. As part of the regional strategy to improve family caregivers’ health, case manager nurses use this database to identify family caregivers of people with different levels of dependency and different conditions. Family caregivers of people diagnosed with advanced AD or any other disease were excluded. In order to verify the stage of AD at which the person was, their clinical histories were consulted. The stage of AD was identified with standardized tests performed by the general practitioner and/or neurologist, according to international guidelines. Out of a total of 1276 relatives included in the database, 507 family caregivers of persons with mild or moderate AD were identified after excluding those who cared for persons in the late stage of AD or other diseases. The sample calculation determined that 255 participants were required to complete the study in order to achieve a 95% confidence level, 3% precision and 7% proportion [8].

### 2.3. Instruments

The data collection sheet designed included: Caregivers’ sociodemographic characteristics (age, gender, degree of kinship, and level of education), persons’ sociodemographic characteristics (age), and variables related to care (living in the person’s household, time spent caring for the person, availability of social, and healthcare support and knowledge of AD).

The Goldberg General Health Questionnaire (GHQ-28) [35], adapted to the Spanish population by Lobo, Pérez-Echeverría, and Artal (1986), was used [36]. This instrument measures caregivers’ ‘perceived health’ and ‘emotional well-being.’ The higher the score, the worse ‘perceived health’ is. The Spanish GHQ-28 (SGHQ-28) is comprised of 28 items divided into four subscales of seven items each (somatic symptoms, anxiety and insomnia, social dysfunction and severe depression) and four Likert-type responses per item. The score for each item is 0 = Not at all, 0 = No more than usual, 1 = More than usual, and 1 = Much more than usual. The GHQ-28 detects non-psychotic mental health problems of new onset, with a 5/6 cut-off point [37]. The GHQ-28 had a Cronbach’s α of 0.90, which demonstrated its internal consistency [36].

The Duke-UNC-11 Functional Social Support Questionnaire (DUKE) [38] was also used. The DUKE measures the subjects’ perceived social support. The DUKE is comprised of 11 items with a Likert-type scale to respond, ranging from 1 = “Much less than what I want” to 5 = “As much as I want”. The total score ranges from 11 to 55. Less than 32 points indicates low perceived social support, while a score equal to or greater than 32 is considered normal. This instrument was validated in Spanish family caregivers and was reported to have a Cronbach’s alpha coefficient of 0.89 [39].

The Barthel Index (BI) [40] adapted by Baztán et al. (1993) for the Spanish population was also used [41]. The BI is a 10-item questionnaire that measures the persons’ functional dependency to perform basic activities of their daily lives. Each item has a score that ranges between 0 and 15 points depending on the activity. The total score ranges from 0 (severe dependency) to 100 (autonomy). In terms of internal consistency, the BI’s Cronbach’s alpha was 0.93.

The Neuropsychiatric Inventory Questionnaire (NPI-Q) was also used [42]. The NPI-Q analyzes psychological and behavioral symptoms in persons. The NPI-Q is comprised of 12 items completed by caregivers and divided into three subscales. In the first subscale, the presence of neuropsychiatric disorders is assessed using dichotomous responses (yes = 1, no = 0). In the second subscale, the severity of the neuropsychiatric symptoms is measured with a Likert-type scale (mild = 1, moderate = 2, and severe = 3). In the third subscale, caregivers’ emotional distress is assessed using a Likert-type scale (from 0 = "Not distressing at all" to 5 = "Extreme or very severe"). In each of these subscales, a maximum score of 12, 36 and 60 can be obtained, respectively. The reliability of this instrument in the Spanish population was 0.89 [43].

### 2.4. Design and Procedure

The study was conducted between January and December 2015. The caregivers were contacted by telephone, and data were collected either during a consultation appointment at the healthcare center or during a person’s home visit. The researchers in charge of the data collection were previously instructed by the lead researcher. Permission was obtained from the provincial research ethics committee (Ethics Committee Almería Center/14/02/12). The informed consent of the participants was requested, both verbally and in writing. The principles of the Declaration of Helsinki, the current data protection law, and the participants’ confidentiality and anonymity were respected.

### 2.5. Data Analysis 

The qualitative variables were analyzed using frequencies and averages. The quantitative variables were analyzed with measurements of central tendency. The primary outcome of the study was ’perceived health.’ The secondary explanatory variables were divided into three groups: The sociodemographic characteristics of the caregiver (age, gender, degree of kinship, and level of education), care-related variables (living in the same home as the person, time spent caring for the person, social and healthcare resources available, knowledge of AD, and perceived social support) and variables related to the person and the disease (i.e., age, person’s level of dependency and neuropsychiatric symptoms). Firstly, a bivariate descriptive analysis was conducted with each of the explanatory variables analyzed, and the response variable ’perceived health’ was used as a continuous quantitative variable. The statistical tests used were: Student’s t-test, one-way ANOVA, and Pearson correlation coefficient. Previously, the Kolmogorov–Smirnov and Levene tests were run to verify the normality and homogeneity of the quantitative variables. Subsequently, a multivariate logistic regression model was developed using the set of predictive or explanatory variables. The response variable of the model was ’perceived health.’ According to the score of the instrument, a score higher than 6 was considered indicative of new-onset mental health problems. A forward procedure with the Wald statistic was the method used, calculating the adjusted odds ratios and their confidence intervals corresponding to 0.05. This procedure allowed us to obtain a model with those predictive variables that best explained the outcome variable. The Hosmer–Lemeshow test and the Nagelkerke coefficient of determination (R^2^) determined the model’s goodness-of-fit and the percentage of variance of the response variable explained by the predictor variables. The sensitivity and specificity of the model was calculated, and this allowed the model’s validity to be assessed. The statistical program SPSS v.25 (IBM Corp, Armonk, NY, USA) for windows was used in the analysis.

## 3. Results

### 3.1. Sample Characteristics

A total of 42.4% (*n* = 108) of the family caregivers cared for persons in the early stage of AD (hereafter referred to as early-stage family caregiver), and 57.6% (*n* = 147) looked after persons in the middle stage of AD (hereafter referred to as middle-stage family caregiver). The family caregivers’ average age was 56.54 years (standard deviation (SD) = 13.13) and 54.47 years (SD = 11.71), respectively. The sociodemographic characteristics of the family caregivers and the persons with AD are shown in Table 1.

### 3.2. Bivariate Analyses

#### 3.2.1. Family Caregivers’ Sociodemographic Characteristics and Perceived Health, Depending on the Stage of the Disease.

No significant correlation was observed between ‘age’ and ‘perceived health’ in early-stage family caregivers (*r* = 0.06; *p* = 0.53) or middle-stage family caregivers (*r* = 0.07; *p* = 0.34). Amongst early-stage family caregivers, the average ‘perceived health’ scores were higher in women than in men. However, these differences were not significant (*t* = −1.56, *p* = 0.12). In middle-stage family caregivers, the average ‘perceived health’ scores were higher in women compared to men, but no significant differences were found between both groups (*t* = −0.79; *p* = 0.43). In both early-stage and middle-stage family caregivers, persons’ spouses showed worse ‘perceived health’ than persons’ children and other relatives. However, significant differences were only found between the different degrees of kinship amongst middle-stage family caregivers (*F* = 3.34, *p* = 0.03). Individuals who had completed primary education showed the worst results in ‘perceived health’ amongst early-stage family caregivers. Conversely, individuals who had not completed any studies were the ones scoring higher on ‘perceived health’ amongst middle-stage family caregivers. Nonetheless, no significant differences were found between the different groups (no studies, primary, secondary and university), neither amongst early-stage family caregivers (*F* = 0.66; *p* = 0.57) nor middle-stage family caregivers (*F* = 1.32, *p* = 0.26) (Table 2).

#### 3.2.2. Variables Related to Caregiving and Perceived Health, Depending on the Stage of the Disease. 

As shown in Table 3, individuals who lived in the same home as the person diagnosed with AD obtained significantly higher mean scores than those who did not live with them, amongst both early-stage family caregivers (*t* = −3.88; *p* ˂ 0.01) and middle-stage family caregivers (*t* = −4.95, *p* = 0.00). In addition, a negative and significant correlation was observed between ‘perceived social support’ and ‘perceived health’ in early-stage family caregivers (*r* = −0.21; *p* = 0.028) and middle-stage family caregivers (*r* = −0.31; *p* ˂ 0.01). This is, the higher the perveived social support, the lower the scores on the GHQ-28, which means better perceived health. For all the other variables (i.e., time spent caring for the person, social and healthcare resources available, and perceived social support), the results did not show significant differences for any of the groups (early-stage and middle-stage family caregivers).

#### 3.2.3. Variables Related to the Person and Perceived Health, Depending on The Stage of the Disease.

Amongst early-stage family caregivers, ‘perceived health’ correlated significantly and positively with the ‘severity of the person’s neuropsychiatric symptoms’ (*r* = 0.22; *p* = 0.05) and with the ‘caregiver’s emotional distress in response to the person’s neuropsychiatric symptoms’ (*r* = 0.22; *p* = 0.05). The correlation was significantly negative between ‘perceived health’ and ‘person’s level of dependency’ (*r* = −0.24, *p* = 0.05). Amongst middle-stage family caregivers, a significant and positive correlation was observed between ‘perceived health’ and ‘presence of neuropsychiatric symptoms’ (*r* = 0.27, *p* = 0.01), ‘severity of the person’s neuropsychiatric symptoms’ (*r* = 0.32, *p* = 0.01) and ‘caregiver’s emotional distress in response to the person’s neuropsychiatric symptoms’ (*r* = 0.029; *p* = 0.01). These results show that when both the person’s neuropsychiatric symptoms severity and the caregiver’s emotional distress in response to these symptoms increased, caregivers’ perceived health decreased. (Table 4).

### 3.3. Multivariate Logistic Regression Model 

In the multivariate logistic regression model, the association between the predictor variables (sociodemographic, care-related and person-related) and the presence of new-onset mental health problems were analyzed. In order to perform this analysis, the response variable ‘new-onset mental health problems’ was divided into two categories: 1) ‘Presence of new-onset mental health problems’ and 2) ‘no presence of new-onset mental health problems.’

Amongst early-stage family caregivers, the percentage of participants with new-onset mental health problems was 46.3% (*n* = 50). Table 5 shows how men have 80% lower risk of suffering from new-onset mental health problems (*OR* = 0.20; *p* ˂ 0.05); therefore, this is a protective factor when compared to being a woman. Furthermore, a ‘caregiver’s emotional distress caused in response to the person’s neuropsychiatric symptoms’ makes the caregiver have a greater risk (8%) of suffering new-onset mental health problems (*OR* = 1.08; *p* ˂ 0.05).

A total of 30.9% of the ‘perceived health’ variance detected amongst early-stage family caregivers was explained by the developed model (Nagelkerke R^2^ = 0.309). The Hosmer and Lemeshow test measured the goodness-of-fit of the model (*X*^2^ = 10.27; *df* = 8; *p* = 0.24). Lastly, the model had 70% sensitivity and 75.4% specificity.

Amongst middle-stage family caregivers, the presence of new-onset mental health problems was detected in 61.9% (*n* = 91) of the participants. As shown in Table 6, the risk of suffering new-onset mental health problems is 42% higher in caregivers who do not have social and healthcare support when compared to those who do so (*OR* = 4.43; *p* ˂ 0.05). Likewise, the severity of the person’s neuropsychiatric symptoms increases the probability of suffering new-onset mental problems by 11% (*OR* = 1.11; *p* ˂ 0.05). On the other hand, family caregivers with greater ‘perceived social support’ have a 6% less probability of developing new-onset mental health problems (*OR* = 0.94; *p* ˂ 0.05). Another protective factor for middle-stage family caregivers is not living in the same household as the person, which in turn reduces the probability of developing new-onset mental health problems by 82% (*OR* = 0.18; *p* ˂ 0.05).

The Nagelkerke coefficient of determination (R^2^) determined that 38.2% of the variance of the response variable was explained by the logistic regression model developed for middle-stage family caregivers. The Hosmer and Lemeshow test established an acceptable goodness-of-fit (*X*^2^ = 13.15; *df* = 8; *p* = 0.10). Furthermore, the model had 83.5% sensitivity and 60.7% specificity.

## 4. Discussion

In this study, a series of variables related to perceived health and presence of new-onset mental health problems in family caregivers were analyzed that allowed us to create a predictive model that could explain the studied phenomenon. Three groups of variables were studied: The sociodemographic characteristics of the caregiver, variables related to caregiving and variables related to the patient and the disease.

The sociodemographic characteristics of this study’s sample were similar to previously-reported ones [14,15,16]. Mainly, family caregivers were middle-age females, patients’ daughters or partners and with low academic backgrounds. The degree of kinship is a sociodemographic variable that can influence middle-stage family caregivers’ perceived health. Concurring with other studies’ results [24], persons’ spouses and daughters are more likely to suffer new-onset mental health problems that other degree of kinship. The moral obligation assumed by most people with a higher degree of consanguinity, together with the responsibility of having to physically and emotionally care for somebody else, could be the cause of the psychological distress that family caregivers of people diagnosed with mild and moderate AD suffer [15]. Together with the degree of kinship, the family caregiver’s gender emerges as a risk factor for suffering new-onset mental health problems for only early-stage caregivers. Being a woman increases the risk of suffering certain mental health problems when compared to men. In fact, other studies have shown a significant prevalence of depression and psychosomatic symptoms amongst female family caregivers [44]. These results may be explained by the type of coping strategy that women use when faced with stressful situations as family caregivers. It has been suggested that female family caregivers often use a coping mechanism that aims to regulate their emotions rather than directly addressing the source of those feelings [45].

Regarding the influence of care-related variables on caregivers’ measured outcomes, this study found that living in the same house as the person negatively influences caregivers’ perceived health in both early-stage and middle-stage family caregivers. Furthermore, and concurring with other studies’ results [29,46], it has been found that not living in the same home as the person becomes a protective factor with regard to developing new-onset mental health problems amongst middle-stage family caregivers. In addition, caregivers’ perceived social support and instrumental support has been found to influence middle-stage family caregivers’ perceived health. Similar to other studies, the perception of social support acts as a modulator to avoid overloading caregivers [25]. In fact, those family caregivers who seek social support experience less emotional reactivity to the daily stressful events they have to face [34]. Han et al. (2014) determined that the emotional support and social interactions perceived by family caregivers affect their psychological burden more than the availability of social and healthcare resources (i.e., instrumental support) [47]. This could justify why family caregivers’ perceived social support should be carefully assessed from an early stage of AD disease and nursing interventions should be orientated towards ensuring that family caregivers have adequate social support.

Regarding the influence of person-related and disease-related variables on caregivers’ measured outcomes, the present study found that the emotional distress that family caregivers suffer as a consequence of the person’s neuropsychiatric symptoms, together with the level of severity of the person’s neuropsychiatric symptoms, negatively affect early-stage and middle-stage family caregivers’ perceived health. On the one hand, the multivariate logistic regression model developed in this study suggests that caregivers’ emotional distress produced by the person’s neuropsychiatric symptoms is an independent risk factor for early-stage family caregivers to develop new-onset mental health problems. On the other hand, the multivariate logistic regression model developed to explain the variables that influence the appearance of new-onset mental health problems amongst middle-stage family caregivers found that both the severity and intensity of persons’ neuropsychiatric symptoms were independent risk factors for developing such problems. These findings confirm the importance and severity of persons’ neuropsychiatric symptoms and emotional distress that is produced in early-stage and middle-stage family caregivers. It has already been suggested that neuropsychiatric symptoms amongst persons with AD have a negative impact on both the caregivers’ mental health [48] and the expenditure of healthcare systems [49]. It is also known that family caregivers’ burden can worsen the person-caregiver relationship, which can in turn increase the severity of persons’ neuropsychiatric symptoms [10]. These results may indicate the need for designing and implementing intervention strategies in emotional regulation that allow family caregivers to adapt to the person’s behavior disorders from an early stage of AD [16]. Lastly, a persons’ level of dependency for performing basic activities of their daily lives is another variable that, together with behavioral disorders, produces a great overload in family caregivers of people diagnosed with AD [27,28]. Concurring with other studies [25], the person’s level of dependency for performing basic activities of their daily life has been found to negatively affect early-stage family caregivers’ perceived health. This could be explained by the fact that while early-stage family caregivers are more concerned with the loss of their relative’s functional capacity, middle-stage family caregivers are more concerned about the person’s behavioral problems [46].

This study has a series of limitations. Firstly, since the study followed a cross-sectional observational design, the risk factors analyzed were prognostic indicators and did not establish a cause–effect relationship. A longitudinal study would be needed in order to determine the strength of the association between the variables that are related to family caregivers’ perceived health and the presence of new-onset mental health problems. Caregiver burden might have a considerable impact on perceived health, but in this study, it did not have a measure. Secondly, the study sample was comprised of caregivers with specific sociodemographic characteristics. and this does not allow for the generalization of this study’s results. Thirdly, perceived health and the presence of new-onset mental health problems were studied in general, not delving deeper into more specific mental disorders such as severe depression or psychosomatic symptoms. Fourthly, although this research focused on Alzheimer’s disease, it may be that people diagnosed with Alzheimer’s disease have other comorbidities, and this could have had an impact on caregivers’ perceived health. Lastly, data were collected through self-administered questionnaires, and it was not possible to guarantee the complete elimination of social desirability bias.

## 5. Conclusions

Perceived health amongst family caregivers of people with mild AD is related to: Perceived social support, living in the same house as the person with AD, the person’s level of dependency to perform basic activities of daily life, the severity of the person’s neuropsychiatric symptoms, and the emotional distress they cause to the family caregiver. The predictive model suggests that caregivers’ gender and emotional distress are independent risk factors for early-stage family caregivers to develop new-onset mental health problems. Therefore, medical and nursing interventions should be aimed at improving coping mechanisms and emotional regulation in family caregivers of people with mild AD. In family caregivers of people with moderate AD, perceived health is influenced by: The degree of kinship, living in the same house as the person with AD, the severity of the person’s neuropsychiatric symptoms, and the emotional distress they cause to caregivers. Perceived social support and the availability of social and healthcare resources also influence middle-stage family caregivers’ perceived health. Instrumental support is essential for middle-stage family caregivers, and nursing interventions should be directed towards fostering their adaptation to the new caregiving demands that arise with the progression of AD. Future research could design specific intervention programs for family caregivers of people with AD. These programs should include effective strategies to target the variables that specifically affect family caregivers’ perceived health when looking after people diagnosed with mild and moderate AD. In addition, the short-term and long-term benefits of these interventions could be analyzed in terms of emotional well-being and the presence of mental health problems in family caregivers of people with mild and moderate AD.

## Figures and Tables

**Table 1:** Sociodemographic characteristics of the family caregivers and the persons with Alzheimer’s disease (AD).

Characteristics	Early-Stage Family Caregivers	Middle-Stage Family Caregivers
Family Caregiver		
Gender	% (*n*)	% (*n*)
Female	81.5 (88)	88.4 (130)
Male	18.5 (20)	11.6 (17)
Level of education	% (*n*)	% (*n*)
No studies	23 (21.3)	19 (12.9)
Primary	42 (38.9)	65 (44.2)
Secondary	30 (27.8)	41 (27.9)
University	13 (12)	22 (15)
Degree of kinship	% (*n*)	% (*n*)
Other kin	10.1 (11).	8.8 (13)
Son/daughter	55.6 (60)	70.1 (103)
Spouse	34.3 (37)	21.1 (31)
Living in same household	% (*n*)	% (*n*)
Yes	43.5 (47)	36.1 (53)
No	56.5 (61)	36.9 (94)
Time spent caring for the patient	% (*n*)	% (*n*)
Less than 2 years	38 (41)	39 (26.5)
2 to 5 years	43.5 (47)	69 (46.9)
More than 5 years	18.5 (20)	39 (26.5)
Social and healthcare resources	% (*n*)	% (*n*)
Yes	66.7 (72)	117 (79.6)
No	33.3 (36)	30 (20.4)
Person with AD		
Gender	% (*n*)	% (*n*)
Female	55.6 (60)	60.5 (89)
Male	44.4 (48)	39.5 (58)
	M (SD)	M (SD)
Age	76.57 (8.19)	80.04 (6.27)
Dependency (BI)	74,77 (23.07)	49.35 (27.51)
NPI-Q presence	6.86 (2.34)	7.97 (2.26)
NPI-Q severity	16.61 (6.9)	19.61 (6.61)
NPI-Q stress	22.84 (9.96)	26.81 (9.48)

Note: M = Mean; SD = Standard deviation; BI = Barthel Index; NPI-Q = Neuropsychiatric Inventory Questionnaire.

**Table 2:** Family caregivers’ sociodemographic characteristics and perceived health, depending on the stage of the disease.

Characteristics	Early-Stage Family Caregivers		Middle-Stage Family Caregivers	
	M (SD)	*p*	M(SD)	*p*
Age	56.54 (7.21)	0.53 ^c^	54.47 (7.97)	0.34 ^c^
Gender				
Female	7.69 (6.91)	0.12 ^a^	8.12 (6.34)	0.43 ^a^
Male	5.1 (5.65)		6.82 (6.14)	
Degree of kinship				
Other kin	5.34 (5.22)	0.07 ^b^	4.46 (5.53)	0.03 ^b^*
Son/daughter	6.3 (6.51)		7.87 (6.53)	
Spouse	9.24 (7.2)		9.74 (6.53)	
Level of education				
No studies	7.74 (6.73)	0.57 ^b^	9.84 (7.08)	0.26 ^b^
Primary	8.07 (6.59)		8.03 (6.26)	
Secondary	6.13 (7.55)		6.59 (6.12)	
VarUniversity	6 (5.43)		8.73 (5.99)	

Note: M = Mean; SD = Standard deviation; *p* = Level signification ^a^ = Student’s *t*-test; ^b^ = One-way ANOVA; ^c^ = Pearson correlation; * The correlation is significant at the 0.05 level.

**Table 3:** Variables related to caregiving and perceived health, depending on the stage of the disease.

Variables	Early-Stage Family Caregivers		Middle-Stage Family Caregivers	
	M (SD)	*p*	M (SD)	*p*
Living in same household				
Yes	10 (7.2)	˂0.01 ^a^*	9.74 (5.26)	˂0.01 ^a^*
No	5.07 (5.51)		4.81 (5.7)	
Time spent caring for the patient				
Less than 2 years	6.15 (6.9)	0.42 ^b^	8.28 (6.09)	0.82 ^b^
2–5 years	8.04 (7.16)		7.62 (6.04)	
More than 5 years	7.45 (6.9)		8.26 (7.10)	
Social and healthcare resources				
Yes	7.17 (7.13)	0.91 ^a^	7.4 (6.23)	0.032 ^a^*
No	7.31 (6.01)		10.17 (6.27)	
Knowledge of caregiving	5.64 (2.12)	0.34 ^c^	5.64 (2.04)	0.53 ^c^
Perceived social support (DUKE)	36.65 (10.03)	0.028 ^c^*	35.06 (9.96)	˂0.01 ^c^**

Note: M = Mean; SD = Standard deviation; *p* = Level signification ^a^ = Student’s *t*-test; ^b^ = One-way ANOVA; ^c^ = Pearson correlation; * The correlation is significant at the 0.05 level; ** The correlation is significant at the 0.01 level.

**Table 4:** Correlations between variables related to the patient and perceived health, depending on the stage of the disease.

Variables	Variables		Early-Stage Family Caregivers				Middle-Stage Family Caregivers				
			NPI-Q			BI		NPI-Q			BI
		Age	Presence	Severity	Stress	Dependency	Age	Presence	Severity	Stress	Dependency
GHQ-28	Perceived health	−0.19	0.16	0.22 *	0.22 *	−0.24 *	−0.13	0.27 **	0.32 **	0.29 **	0.13
	Age		0.07	0.7	0.6	−0.32 **		−0.06	−0.07	−0.07	−0.32 **
NPI-Q	Presence			0.95 **	0.91 **	−0.22 *			0.94 **	0.92 **	0.04
	Severity				0.98 **	0.22 *				0.98 **	−0.07
	Stress					−0.20 *					0.06

Note: NPI-Q = Neuropsychiatric Inventory Questionnaire; BI = Barthel Index; GHQ-28 = Goldberg General Health Questionnaire; * Correlation is significant at the 0.05 level; ** Correlation is significant at the 0.01 level.

**Table 5:** Predictors of perceived health amongst early-stage family caregivers: Multivariate logistic regression.

Variables	β	S.E.	Wald	*p*	aOR	CI 95% for OR
Gender						
Woman						
Man	−1.60	−0.65	5.98	0.01	0.20	0.05–0.72
Degree of kinship						
Other kin						
Son/daughter	−0.34	−0.74	0.21	0.64	0.70	0.16–3.06
Spouse	1.03	0.78	1.72	0.18	2.80	0.60–13.06
Living in same household						
Yes						
No	−0.85	0.45	3.55	0.05	0.42	0.17–1.03
NPI-Q stress	0.08	0.02	10.40	˂0.01	1.08	1.03–1.13
Constant		–1.38	0.98	1.96	0.16	0.25

Note: β = Regression coefficient; S.E. = Standard error; OR = Adjusted odds ratio; CI = Confidence interval.

**Table 6:** Predictors of perceived health amongst middle-stage family caregivers: Multivariate logistic regression.

Factors	β	S.E.	Wald	*p*	aOR	CI 95% for OR
Social and healthcare resources						
Yes						
No	1.49	0.59	6.35	0.01	4.43	1.39–14.13
Perceived social support (DUKE)	−0.06	0.02	7.75	˂0.01	0.94	0.90–0.98
Living in same household						
Yes						
No	−1.66	0.43	14.57	˂0.01	0.18	0.08–0.44
NPI-Q severity	0.10	0.03	9.78	˂0.01	1.11	1.04–1.18
Constant		1.01	1.05	0.92	0.33	2.76

Note: β = Regression coefficient; S.E. = Standard error; OR = Adjusted odds ratio; CI = Confidence interval.
